# Inhibitory effects of subcutaneous tumors in nude mice mediated by low-frequency ultrasound and microbubbles

**DOI:** 10.3892/ol.2014.1934

**Published:** 2014-03-04

**Authors:** ZHI-YONG SHEN, E. SHEN, XUE-HONG DIAO, WEN-KUN BAI, MIN-XIA ZENG, YAN YAN LUAN, SHU-LIANG NAN, YAN-DUAN LIN, CONG WEI, LI CHEN, DI SUN, BING HU

**Affiliations:** 1 Department of Ultrasound in Medicine, Shanghai Institute of Ultrasound in Medicine, Sixth People’s Hospital Affiliated to Shanghai Jiaotong University, Shanghai Jiaotong University School of Medicine, Shanghai 200233, P.R. China; 2 Department of Radiology, Nantong Tumor Hospital Affiliated to Nantong University, Nantong, Jiangsu 226361, P.R. China

**Keywords:** ultrasound, low frequency, microbubbles, cavitation

## Abstract

The aim of the present study was to investigate the sonication effects of 21-kHz ultrasound (US) with microbubbles (MBs) on the subcutaneous prostate tumors of nude mice. In total, 15 tumor-bearing nude mice were divided into three groups: The control group, the low-frequency US group and the US+MB group. The MBs used were from US contrast agent SonoVue. The parameters of the US were as follows: 21 kHz, 26 mW/cm^2^ and a 40% duty cycle (2 sec on, 3 sec off) for 3 min, once every other day for 2 weeks. Color Doppler flow imaging, hematoxylin and eosin (HE) staining, immunoblotting and transmission electron microscopy (TEM) were used to evaluate the results. Following 2 weeks of treatment, the blood flow signal disappeared in the US+MB group only, and the tumor size was smaller when compared with the control and US groups. For the immunoblotting, the intensity of cyclooxygenase-2 and vascular endothelial growth factor in the US+MB group was lower compared with the other two groups. Tumor necrosis was present and the nucleus disappeared upon HE staining in the US+MB group. Upon TEM analysis, increased cytoplasmic vacuolation and dilatation of the perinuclear cisternae of the tumor cells were found in the US+MB group. In the control and US groups, the tumors had intact vascular endothelia and vessel lumens. However, lumen occlusion of the vessels was observed in the US+MB group. In conclusion, 21-kHz low-intensity US with MBs may result in vessel occlusion and growth inhibitory effects in the subcutaneous tumors of nude mice.

## Introduction

Low-frequency ultrasound (US) is usually referred to as ultrasound with frequencies in the range of 20–100 kHz (1–2). The relatively long wavelengths indicate that the low-frequency ultrasonic wave is affected by larger obstacles than high-frequency US in its passage through the propagation medium. This results in a lower spatial resolution, and the low-frequency range is therefore less useful for diagnostic medical applications. However, it has a diverse set of industrial and medical applications (2). In fact, the industrial applications of US mainly occupy this frequency range (3–4). The bioeffects of low-frequency US include thermal and cavitational effects and other ‘mechanical’ effects, including acoustic micro-streaming and radiation forces, among which cavitation is generally accepted as the most significant mechanism (5).

As a broad definition, acoustic cavitation is the process by which any of the following occurs: i) The pulsation or growth of small gas bubbles already present in a liquid; ii) the formation of gas bubbles in the bulk or on nuclei as a result of acoustic pressure variations; or iii) other types of growth, splitting or interactions of gas bubbles in solution caused by acoustic pressure oscillations (1). Acoustic cavitation is further divided into stable and transient types. The pulsation of cavitation bubbles over numerous acoustic pressure cycles without collapse is known as stable cavitation (6), whereas transient cavitation is rapid and uncontrolled bubble growth over several pressure cycles, leading to the eventual collapse into smaller bubbles (1).

For inertial decavitation, bubbles have more time to grow by rectified diffusion in the expansion half cycle when using a lower frequency wave. Therefore, it can be hypothesized that at lower US frequencies, transient cavitation will have a more significant effect (2,7).

The permeability of individual cells for an improved delivery of drugs and genes can be achieved by inertial cavitation (collapsing bubbles). This process is known as sonoporation (8); sound energy is used to create pores and as a result enhance the permeability of plasma membranes.

To further our understanding of cavitation-based mechanisms, to optimize intracellular uptake, to control bioeffects and to advance techniques for clinical applications, previous US-enhanced delivery studies have focused on delivery into *in vitro* cells in suspension (9–10) and more recently into tissues of *in vivo* animal models. The threshold for the onset of *in vivo* cavitation depends on the presence of cavitation nuclei of appropriate size (11). The natural occurrence of bubbles has only been observed in the digestive and respiratory tracts, but not elsewhere for *in vivo* blood (12). Without cavitation nuclei in the blood, the occurrence of cavitation at low acoustic pressures *in vivo* is dependent on the injection of stabilized bubbles. The objective of the present study was to associate the bioeffects of tumor-bearing nude mice exposed by 21-kHz US and contrast agent bubbles injected from the tail veins of mice.

## Materials and methods

### Animal protocol

In total, 25 male nude mice, aged 4 weeks old and weighing 15–18 g, were purchased from the Animal Center of the Shanghai Institute of Chinese Academy of Science (Shanghai, China). All mice were treated and housed according to approved guidelines (Guidelines for the Care and Use of Laboratory Animals). Following anesthesia by intraperitoneal injection of 0.004 g ketamine, the mice were secured to a superclean bench according to the principles of aseptic procedures. Following local sterilization, each mouse was then subcutaneously inoculated with 2×10^6^ cells from the DU145 cell line into the flank. The mice continued to be raised under specified pathogen-free conditions subsequent to the procedure, and were observed at 2-day intervals. Experiments were initiated 2 weeks later, once the tumors had reached a size of 5–8 mm. This study was approved by the ethics committee of Shanghai Jiao Tong University Affliated Sixth Hospital, (Shanghai, China).

### Experimental groupings for tumor therapy and experimental protocol

**Figure 1:**
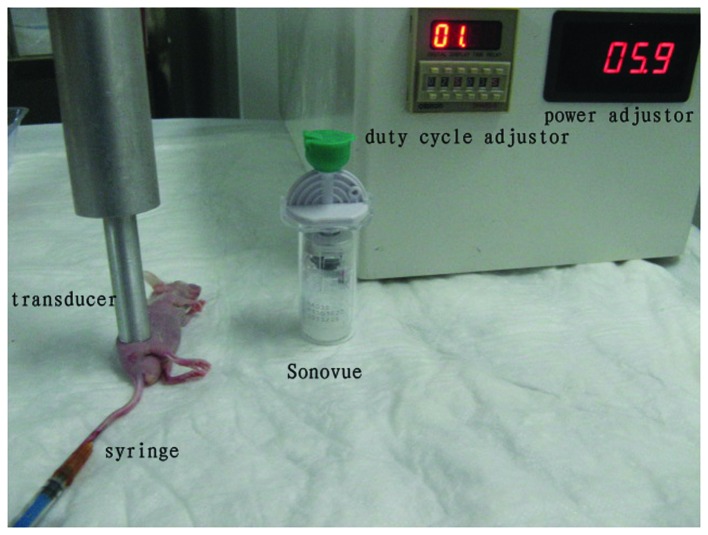
Therapy apparatus for low-frequency US and experimental set-up. US, ultrasound.

In total, 15 nude mice, each with a subcutaneous tumor of 6 mm in size, were randomly divided into three groups, with five mice in each group. These groups were as follows: The A group, negative control (sham treatment); the B group, low-frequency US; and the C group, US+microbubbles (MB). The MBs used were from a US contrast agent (SonoVue, Bracco Imaging SpA, Milan, Italy). The mice were anesthetized by intraperitoneal injection of 0.3 ml 1% pentobarbital sodium. Following successful anesthesia, the tumor xenografts were subsequently sonicated using a transducer (Fig. 1), manufactured in the Shanghai Institute of Ultrasound in Medicine at Shanghai Jiaotong University (Shanghai, China), and placed on the skin with contact gel (Aquasonic 100; Parker Laboratories, Inc., Fairfield, NJ, USA). The diameter of the therapeutic US transducer was ~13 mm, which covered the entire tumor. Low-frequency US parameters were set at 21 kHz, 26 mW/cm^2^, a duty cycle of 40% (2 sec on, 3 sec off) and a duration of 3 min once every other day for two weeks, which was the same as our previous study (13).

### Color Doppler flow imaging (CDFI)

CDFI is a useful tool for assessing tumor neovascularity and also for monitoring anti-angiogenic therapies. At the initiation (0 weeks) and completion (2 weeks) of the present experiment, the subcutaneous prostate cancer of the nude mice was examined by CDFI. CDFI images of the tumors were obtained using a Mylab90 instrument (Esaote, Genoa, Italy) handled by an experienced examiner. The frequency of the probe used was 15 MHz. For the present study, the sensitivity of the instrument was set at a low velocity in order to display a low blood flow signal. Only the intratumoral blood signal was evaluated.

### Histological examination

At the end of the experiment, the tumor size of each mouse was calculated using a calibrator, and then the mice were euthanized and the tumors collected, fixed and embedded in paraffin. Sections were taken from the middle region of each tumor, followed by hematoxylin and eosin (HE) staining and subsequent light microscopy. A histopathologist blinded to the study evaluated the microscopy findings.

### Western blotting assays

The mice were sacrificed at the end of the experiment and the tumors were collected. The detection of the protein expression of cyclooxygenase (COX)-2 and vascular endothelial growth factor (VEGF) was assessed using the western blot assay. The following primary antibodies were used: goat polyclonal anti-COX-2 and goat polyclonal anti-VEGFB antibodies (1:500 dilution; Santa Cruz Biotechnology, Inc., Santa Cruz, CA, USA). The cancerous tissues were lysed in radioimmunoprecipitation assay buffer (150 mM NaCl, 100 mM Tris-HCl, 1% Tween-20, 1% sodium deoxycholate and 0.1% SDS) with 0.5 mM EDTA, 1 mM phenylmethanesulfonyl fluoride, 10 μg/ml aprotinin and 1 μg/ml pepstatin. Proteins were subjected to SDS-PAGE and transferred to polyvinylidene fluoride membranes, which were then treated with the primary and secondary antibodies (goat anti-mouse IgG horseradish peroxidase-conjugated, 1:500 dilution, Santa Cruz Biotechnology, Inc.). Visualization was carried out using an enhanced chemiluminescence method (Amersham Bioscience, Boston, MA, USA). Subsequent to being stripped, the membranes were reprobed with β-actin (Oncogene, CN Biosciences, Inc., Darmastadt, Germany). The proteins were quantified using an Image Acquisition and Analysis System (Ultra-Violet Products, Upland, CA, USA).

### Transmission electron microscopy (TEM)

For the TEM analysis, each tumor sample (~1 mm^3^) was fixed in 2% glutaraldehyde and phosphate-buffered saline (PBS) for 2 h at 4°C, and then washed twice with PBS buffer for 10 min. Following treatment with 1% osmium tetroxide in PBS, the specimens were fixed in 4°C for 2 h and dehydrated with 30%, 50% and then 70% ethanol three times each for a duration of 10 min. The samples were then embedded in propylene oxide for 2 h and stained with lead citrate E. Finally, subsequent to sectioning, the specimens were examined using TEM (Philips CM-120; Philips, Eindhoven, The Netherlands).

### Statistical analysis

The statistical analysis was performed using SPSS, version 11.0 (SPSS Inc., Chicago, IL, USA). Student’s t-test was used to make a statistical comparison between groups. All testing was carried out using Prism 3.0 (GraphPad, San Diego, CA, USA). Error bars represent the standard error above the mean. P<0.05 was considered to indicate a statistically significant difference.

## Results

### CDFI

**Figure 2:**
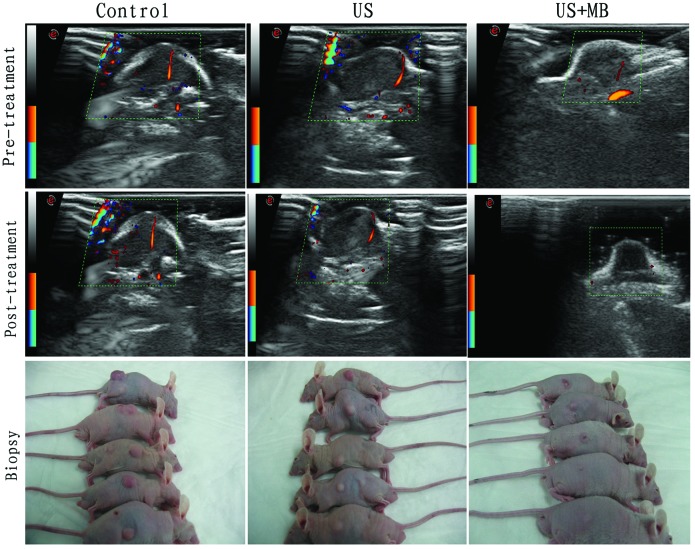
CDFI prior to and following treatment in the control, US and the US+MB group. Following 2 weeks of treatment, the blood flow signal in the US+MB group disappeared, and the tumor size was smaller when compared with the other 2 groups. CDFI, color Doppler flow imaging; US, ultrasound; MB, microbubble.

Prior to the treatment, CDFI demonstrated a blood flow signal within all the tumors of the three groups. In the US+MB group only, the blood flow signal disappeared following 2 weeks of treatment, while in the control and US group, the flow signal in the tumors remained (Fig. 2).

### Tumor size calculation

**Figure 3:**
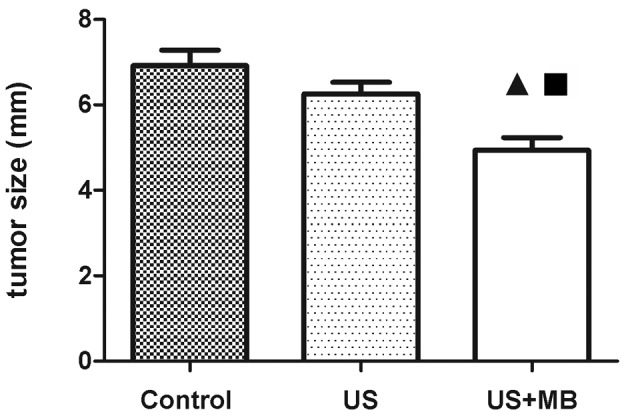
Tumor sizes of the 3 groups after 2 weeks of treatment. Tumor size in the US+MB group was smaller compared with the US and control groups. ^▲^P<0.05 vs. control group. ^■^P<0.05 vs. US group. US, ultrasound; MB, microbubble.

The tumor size of the three groups is manifested in Figs. 2 and 3. There were significant differences in tumor size among the three groups, as determined using the ANOVA test; F=8.418 and P=0.0052. There was a significant difference between the US+MB group and the control and US groups, with t=3.804 and P=0.0052, and t=3.117 and P=0.0143, respectively (Figs. 2 and 3).

### Western blotting assays results

The mean intensity values for COX-2 in the vascular endothelial cells and cytoplasm in the control, US and US+MB groups were 1.203±0.074, 1.114±0.036 and 0.4783±0.114, respectively. There was a significant difference between the US+MB group and the control and US groups, with t=5.338 and P=0.0007, and t=5.303 and P=0.0007, respectively.

**Figure 4:**
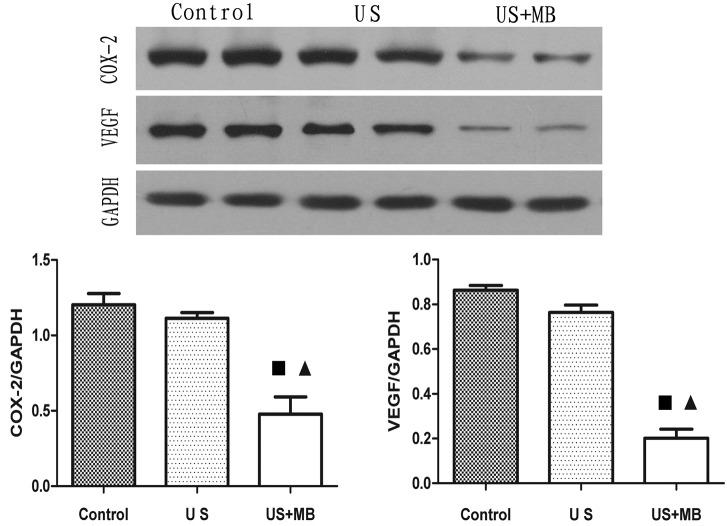
Immunoblotting results. In the US+MB group, the staining intensity of COX-2 and VEGF was clearly decreased compared with the other two groups (P<0.05). ^▲^P<0.05 vs. control group; ^■^P<0.05 vs. US group. US, ultrasound; MB, microbubbles; COX-2, cyclooxygenase 2; VEGF, vascular endothelial growth factor.

The mean intensity values for VEGF in the vascular endothelial cells and cytoplasm in the control, US and US+MB groups were recorded as 0.863±0.021, 0.764±0.033 and 0.202±0.041, respectively. There was a significant difference between the US+MB group and the control and US groups, with t=14.59 and P<0.0001, and t=10.8 and P<0.0001, respectively (Fig. 4).

### HE staining results

**Figure 5:**
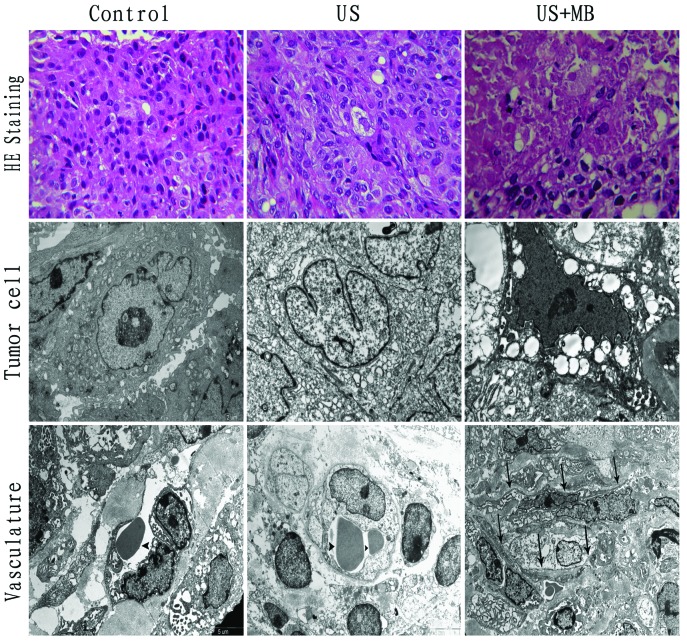
HE results demonstrating tumor necrosis and the disappearance of the nuclei in the US+MB group, the tumor cells of the 3 groups by TEM at 2 weeks and the increased cytoplasmic vacuolation and dilatation of the perinuclear cisternae in the US+MB group. Normal tumor cells are observed in the control and US groups. Microvessels in the control (arrowhead) and US (arrowhead) groups exhibit intact vascular lumens and normal erythrocytes in the vessels; lumen occlusion (arrows) is observed in the US+MB group. HE, hematoxylin and eosin; US, ultrasound; MB, microbubble; TEM, transmission electron microscopy.

In the control and US groups, the tumor cells were intact, with nuclei that were abnormal, large and deeply stained. However, in the US+MB group, the tumor cells in the exposed area presented with coagulative necrosis and the nuclei disappeared (Fig. 5).

### TEM results

TEM revealed apparent cytoplasmic vacuolation and dilatation of perinuclear cisternae in the tumor cells, and vascular lumen occlusion in the US+MB group. The majority of tumor cells were identified as normal in the other two groups. Intact vascular lumens and normal erythrocytes in the tumor vessels were also found in the control and US groups (Fig. 5).

## Discussion

In this era of US research, several novel applications of US for therapy are undergoing intensive investigation and development. MB-based therapeutic strategies are under study for US-directed and targeted therapy. In the present study, low-frequency, low-energy US aided by stabilized MBs was used for the treatment of nude mouse tumors.

This procedure caused substantial destructive effects on the tumor cells, which was evidenced by coagulation necrosis (Fig. 5) and apparent tumor shrinkage (Figs. 2 and 3) compared with the two other groups. In this strategy, the external US exposure activates MBs as cavitation nuclei in the circulation at a desired site of treatment. The addition of US contrast agents, as a source of cavitation nuclei during exposures, renders cavitation activity more predictable and also lowers the intensity threshold for its onset (14). US fields produce a varying local pressure, which causes gas-filled bubbles to expand and contract due to their high compressibility, and these volumetric oscillations are significant in their effectiveness in therapeutic applications (11). In the present study, tumor destruction was apparently due to inertial cavitation. US combined with MB caused a decrease in tumor growth. It is worth noting that the same exposures without MBs did not cause significant tumor size shrinkage compared with the sham control. Tumor growth inhibition may be the result of US-mediated bioeffects by low-frequency US sonication with MBs.

Angiogenesis is the development of new blood vessels. For the growth of tumors and the formation of metastases, new blood vessels are required. Neovascularization can be identified inside the tumor and in the peritumoral tissue (15). Visualization of tumor vascularity (16) can be demonstrated by CDFI, and it is an established technique for the evaluation of anti-neovascular effects (17). In animal models, CDFI can track the response of tumors to chemotherapy and radiotherapy (17). Prior to the treatment in the present study, the blood flow signals were all identified within the tumors in the control, US and US+MB groups. However, following treatment, the intertumoral flow signal disappeared in the US+MB group, while it remained in the control and the US groups (Fig. 2). It is known that anti-angiogenic agents can inhibit angiogenesis (18). Vascular targeting agents, including drugs and vascular disrupting agents, aim to inhibit new vasculature growth or destroy existing vasculature, respectively. It is possible that a low frequency ultrasound combined with MBs may have specific anti-angiogenic effects.

In order to evaluate the results of the treatment, western blotting assays were used to detect angiogenesis-associated gene proteins, including VEGF and COX-2, in the tumor tissue. VEGF and COX-2 are associated with carcinogenesis due to the stimulation of cell proliferation, the inhibition of apoptosis and the enhancement of angiogenesis (19). The inhibition of VEGF and COX-2 is conceivably an attractive therapeutic target in the treatment of cancer. The results of the present study showed that following treatment, VEGF and COX-2 gene expression in the US+MB group was lower compared with the control and US groups, which was consistent with our previous study (13). Tumor sonication following intravenous injection of MBs could downregulate angiogenesis-associated gene proteins in nude mouse tissues.

Using TEM in the present study, intact vascular lumens and normal erythrocytes were observed in the blood vessels following treatment in the control and US groups (Fig. 5, arrowhead). However, following US treatment in the presence of MBs, degeneration of the endothelial cells and lumen occlusion were observed in these vessels, which indicates that the effect of US+MB is different from that of the other two groups. The tumor cell changes that were observed included cytoplasmic vacuolation and dilatation of the perinuclear cisternae in the US+MB group. US in combination with the contrast agent resulted in apparent damage to the blood vessels and tumor cells in the cancer of the nude mice. The results of the present study, indicate that further study is required into the underlying mechanism responsible for these effects.

MBs, which are artificially augmented cavitation nuclei, play a significant role in the treatment of murine tumors during anti-vasculature therapy. A significant factor that will determine whether cavitation will or will not occur is the available physical space for bubbles to form and grow. The induction of cavitation within intact cells and in the extracellular matrix is difficult (8). However, when a high enough *in vitro* US pressure fields exists, the vasculature possesses injected cavitation nuclei and the required dimensions for the initiation of cavitation. In the US group of the present study, the reason that tumor growth inhibition was not obvious is due to the dearth of cavitation nuclei and a lack of physical space for bubble oscillation and expansion in the tumoral interstitium. The addition of MBs has also been found to decrease the intensity threshold for producing damage in treated vessels (20).

Insonified by low-frequency US pressure, bubbles become unstable, grow, oscillate and collapse in blood vessel fields, and this phenomenon is referred to as the acoustic cavitation bioeffect. Damage to nearby biological cells and structures, including vascular endothelia and vessel lumens, can occur due to the concentration of acoustical energy and its conversion into local mechanical perturbation during cavitation. When US exposes the tumors following an injection of MBs, the interaction of the US beam with the MBs in the blood vessels results in the expansion, oscillation and collapse of the bubbles, followed by vicinal blood vessel distention, invagination and deformation (21–22). Bubble expansion significantly distends the vessel to ~2.7 times its original diameter (21), followed by bubble collapse at the msec phase leading to almost axially symmetric vessel invagination. During invagination, the notch-like shape on the sides of the vessel wall indicate high strains on the vessel wall. Invagination, which generates higher strains on the vessel wall than distention, was commonly observed in the present study when bubbles collapsed near the vessel wall, which pulled the vessel inward toward the lumen. We hypothesize that the cumulative effects of vessel invagination produced by substantial MB fragmentation irradiated by 21-kHz US had a tendency to lead to vascular stenosis, and that the long-term effects of this will eventually lead to vascular occlusion. More in-depth studies are required to prove that this proposed mechanism is conclusive. In the present study, it was observed that the lumen occlusion of a vessel, leading to a decreased blood supply, may be the major reason for the disappearance of the blood flow signal on CDFI, for the decline in COX-2 and VEGF expression in western blotting assays and for the inhibition of tumor growth after a 14-day treatment.

There were certain limitations to the present study. First, there was a lack of a MB-only group. However, it was assumed that the diameter of the bubbles was ~2.5 μm, which was smaller than the red blood cells, but larger than the vascular endothelial gap. Thus, the bubbles seldom penetrated into the tissue spaces. Therefore, in the MB only group, these bubbles had little effect on the vascular endothelia and tumor tissues. Second, potential adverse effects on blood vessels in normal tissues were not investigated in the US+MB group and thus require further exploration in the future.

Synergistic effects were found when combining the low-frequency US exposures with the agents with regard to apparent lumen occlusion of the micro-vessel walls, decreased regulation of VEGF and COX-2 and increased tumor regression of targeted tumors. The mechanisms of the anti-tumor effects are complex and may be mediated by acoustic cavitation. However, *in vivo* cavitation bioeffects were determined by several experimental acoustic parameters, including pressure, exposure time, frequency and MB concentration. Elucidation of the mechanism by which the interactions between the bubbles, low-frequency US wave and blood vessels create these bioeffects is required. This will ultimately be achieved by continuing to collect *in vivo* experimental data, along with continuing to develop appropriate experimental apparatus, which together will enable more efficient optimization of the treatments with regard to the multiple exposure parameters that may be selected. In addition, by having a comprehensive understanding of the way in which the acoustic and physical characteristics of the tissues are involved in these mechanisms, more challenges remain prior to the combination of low-frequency US and MBs becoming a realistic clinical therapy.
